# Quantifying the Hydrophobic Effect per CF_2_ Moiety from Adsorption of Fluorinated Alcohols at the Water/Oil Interface

**DOI:** 10.3390/molecules29071421

**Published:** 2024-03-22

**Authors:** Boyan Peychev, Dimitrinka Arabadzhieva, Ivan L. Minkov, Elena Mileva, Radomir I. Slavchov

**Affiliations:** 1 School of Engineering and Materials Science, Queen Mary University of London, Mile End Road, London E1 4NS, UK; b.peychev@qmul.ac.uk; 2 Rostislaw Kaischew Institute of Physical Chemistry, Bulgarian Academy of Sciences, Acad. G. Bonchev Str., bl. 11, 1113 Sofia, Bulgaria; dimi@ipc.bas.bg (D.A.); minkov.ivan@gmail.com (I.L.M.); mileva@ipc.bas.bg (E.M.); 3 Department of Chemistry, Biochemistry, Physiology, and Pathophysiology, Faculty of Medicine, Sofia University, 1 Koziak Str., 1407 Sofia, Bulgaria

**Keywords:** hydrophobic effect, fluorotelomer alcohol, adsorption model, water-soluble perfluoroalkyl substances, tensiometer

## Abstract

Amphiphilic fluorocarbon substances are a trending topic of research due to their wide range of applications accompanied by an alarming environmental and health impact. In order to predict their fate in the environment, use them more economically, develop new water treatment methods, etc., a better understanding of their physicochemical behavior is required. Their hydrophobicity in water/oil systems is particularly sensitive to one key thermodynamic parameter: the free energy of transfer of a perfluoromethylene group from oil to water. However, for the –CF_2_– moiety, the transfer energy values reported in the literature vary by more than ±25%. Due to the exponential relationship between this energy and the adsorption constants or the partition coefficients, such an uncertainty can lead to orders of magnitude error in the predicted distribution of fluorinated species. We address this problem by presenting an experimental determination of the hydrophobic effect of a –CF_2_– moiety with a greater certainty than currently available. The transfer energy is determined by measuring the interfacial tension of water|hexane for aqueous solutions of short-chained fluorotelomer alcohols. The obtained results for the free energy of transfer of a –CF_2_– moiety from oil to water are $1.68\pm 0.02\times RT_{0}$, $1.75\pm 0.02\times RT_{0}$, and $1.88\pm 0.02\times RT_{0}$ at 288.15 K, 293.15 K, and 303.15 K, respectively.

## 1. Introduction

Fluorocarbons and their derivatives have a number of unique properties that make them valuable in many industrial branches. In fact, in a recent study, Glüge et al. identified almost 300 unique uses for perfluoroalkyl substances [1]. In more than a third of these applications, the perfluoroalkyl substances are used because of their superior surface activity. Due to the larger size of the fluorine atom compared to the hydrogen atom, fluorocarbons are more hydrophobic than hydrocarbons, making fluorosurfactants more effective at reducing the interfacial energy compared to conventional surfactants of similar molecular size [2]. Furthermore, due to the extreme electronegativity and low polarizability of the fluorine atom, fluorocarbons are also oleophobic, allowing for coatings that are simultaneously water- and oil-repellent [2,3]. On the other hand, fluorine-containing organic substances have received much media attention under the umbrella term “forever chemicals” because their chemical-, thermal-, and photo-stability leads to accumulation in the environment. The shorter water-soluble fluorosurfactants, often surface-active products of chemical degradation, have been transported by the water cycle all over the world [4,5,6]. Furthermore, perfluoroalkyl substances have been associated with numerous health hazards [7,8,9]. Thus, there is regulatory action being taken to limit the use of fluorocarbon-based substances [7,10] and a great interest in remediation technologies [7,11,12]. A better understanding of the physicochemical properties of fluorosurfactants could allow for their more efficient use (i.e., use of smaller quantities); could allow for more accurate modeling of their fate in the environment and in the human body or of their interaction with lipid structures [13]; and could aid in the design of separation methods.

In general, fluorocarbon substances are much less studied than their hydrocarbon analogues. For instance, even though separation methods for fluorocarbon substances are a trending topic [7,11,12], their partitioning behavior has not been extensively investigated yet [12]. For perfluoroalkylated substances, the bulk partitioning and the adsorption out of aqueous solutions are driven by the hydrophobic effect, as quantified by the free energy $\Delta \mu_{{\mathrm{CF}}_{2}}$ for transferring the nonpolar moiety –CF_2_– from the hydrophobic phase to the aqueous phase. The values of its aliphatic counterpart, $\Delta \mu_{{\mathrm{CH}}_{2}}$, are well-known for both air and oil phases under a range of conditions [14,15,16,17,18,19,20,21,22] and prove to be very useful for calculating the thermodynamic parameters of homologous series of aliphatic substances, such as partition coefficients, adsorption constants, critical micelle concentrations, etc. In contrast, for the –CF_2_– fragment, the value of the transfer energy between oil and water has not been established with certainty. The reported results for $\Delta \mu_{{\mathrm{CF}}_{2}}$ from oil to water are based on data for the change in the length of the hydrophobic tail ($C_{n}F_{2n+1}$–) of the partition coefficient [23] and on the adsorption constant of fluorinated ionic surfactants [24] (see the discussion in Peychev and Slavchov [25]), and they range from $\Delta \mu_{{\mathrm{CF}}_{2}}=1.6$ to $2.2\times RT_{0}$ (*R* is the universal gas constant, $T_{0}=298.15$ K). This high uncertainty results in at least an order of magnitude uncertainty in the predicted adsorption constants and the oil/water partition coefficients of fluorinated substances.

The main reason for this status quo is that the fluorinated substances that are in common use have long chains and negligible solubility in water. This makes the adsorption out of aqueous solution impossible and the analysis of their partitioning behavior difficult. In contrast, the typical fluorocarbon bioaccumulating pollutants are the shorter water-soluble species, which can be circulated in the water cycle. Furthermore, at least in some cases, the shorter species are more ecotoxic [26]. However, the properties of the water-soluble fluorosurfactants, in particular the nonionic ones, have been scarcely studied. Another reason for the uncertainty in the $\Delta \mu_{{\mathrm{CF}}_{2}}$ estimates is that, unlike their fatty analogues, unbranched perfluorinated chains have two preferred conformations and can transition between the two, as demonstrated by structural studies [27,28,29,30], making $\Delta \mu_{{\mathrm{CF}}_{2}}$ length-dependent.

Perhaps the most precise method of determining $\Delta \mu_{{\mathrm{CF}}_{2}}$ is through the adsorption constant $K_{a}$ for fluorosurfactant adsorbing from a water solution to the water|oil interface. The adsorption constant has an exponential dependence on $\Delta \mu_{{\mathrm{CF}}_{2}}$, i.e., each unit of –CF_2_– added to the hydrophobic tail increases the surface activity of the surfactant by a factor of $\exp(\Delta \mu_{{\mathrm{CF}}_{2}}/RT)$, which is on the order of 5–6, e.g., for ideal solutions, one –CF_2_– decreases sixfold the concentration needed to achieve the same interfacial tension, a variant of Traube’s rule [31]. This dependence allows the determination of $\Delta \mu_{{\mathrm{CF}}_{2}}$ from tensiometric data. Accordingly, in this work, we aim to do exactly that—determine $\Delta \mu_{{\mathrm{CF}}_{2}}$ via tensiometry for homologous series of water-soluble fluorinated nonionic surfactants adsorbing on the water|oil interface. The practical limitation of this approach is that perfluoroalkylated nonionic compounds are very hydrophobic. Perfluorinated chains with as few as five carbon atoms already partition almost completely to the oil phase and, moreover, have exceedingly low solubility in water. On the other hand, the shortest members of a homologous series are known to deviate from Traube’s rule. Thus, no more than a few homologues can be used for the determination, and the result has to be corrected for the expected deviations.

One solution to the low solubility problem is the use of fluorinated ionic surfactants, as done by Mukerjee and Handa [24], who used salts of perfluorinated carboxylic acids. However, using ionic surfactants complicates the interpretation of the results due to the change in the surface charge, the ionic strength effects, and the possibility for hydrolysis at the surface. Moreover, specifically in Mukerjee and Handa’s [24] experiments, only two homologues were studied and only in the infinite dilution region, where impurities, adsorption kinetics, and possible depletion effects may complicate the determination of the equilibrium interfacial tension. These factors increase the uncertainty of the obtained $\Delta \mu_{{\mathrm{CF}}_{2}}$. Instead, here we focus on nonionic surfactants, which both eliminates potential sources of error due to surface charging and makes the analysis simpler.

To achieve the set goal, we collected tensiometric data for the three shortest water-soluble fluorotelomer alcohols of the type $F{\left({\mathrm{CF}}_{2}\right)}_{n}{\mathrm{CH}}_{2}\mathrm{OH}$, with n=1,2,3, adsorbing on the water|hexane interface (see Table 1). The particular choice of alcohols as surfactants and hexane as the oil phase was based on the large amount of previous work done for similar systems, usually with longer oil-soluble homologues; this includes experimental [32,33,34,35,36,37,38], theoretical [25], and simulation studies [39]. Furthermore, we also investigated the temperature dependence of $\Delta \mu_{{\mathrm{CF}}_{2}}$ in the range 288.15–303.15 K.

For the analysis of the tensiometric data, we used the sticky disk adsorption model [40,41] and the molecular thermodynamic model for $K_{a}$ of Ivanov et al. [21,22,41], both of which were shown previously to work well for longer oil-soluble fluorotelomer alcohols [25]. The explicit model for $K_{a}$ accounts for the deviations from Traube’s rule for short tail-lengths, which allows us to determine a more accurate value of $\Delta \mu_{{\mathrm{CF}}_{2}}$.

## 2. Theory

### 2.1. SD Model

The sticky disk (SD) adsorption model was developed to describe fluid monolayers (i.e., nonlocalized adsorption) on soft matter interfaces [40,41]. At its core, it is a hard-disk fluid model based on the scaled particle theory, wherein surfactant molecules occupy a certain hard-disk area α and can move freely on the surface without overlapping [42]. Ivanov’s group also added a 1D sticky potential correction [40] to account for the lateral attraction. This results in an additional attraction parameter β, making the SD model a three-parametric model, i.e., α, β, and the adsorption constant $K_{a}$. Furthermore, it was shown that β implicitly accounts for the depletion attraction effect from the solvent present in the monolayer [43]. The SD model works well for noncohesive and weakly cohesive monolayers, with lateral attraction parameter β<<38, but fails when strong attraction is present β≥38 [21,22]. A comparison of the SD model to other, more popular three-parametric adsorption models, such as those of Frumkin and Van der Waals, shows that the SD model is superior in the sense that all of its parameters correspond to their theoretical definition and can be predicted from independent data, while for the others, they are more or less empirical [22]. We have previously shown that the SD model works very well for fluorotelomer alcohols with 8–12 carbon atoms [25] that are structurally similar but more cohesive than the ones studied here.

The SD equation of state reads:

(1) $$\Pi =RT\frac{R_{\beta }-1}{2\alpha \beta (1-\alpha \Gamma )},\;\mathrm{where}\;R_{\beta }=\sqrt{1+4\beta \frac{\alpha \Gamma }{1-\alpha \Gamma }},$$

and where $\Pi =\gamma_{0}-\gamma$ is interfacial pressure, γ is interfacial tension, $\gamma_{0}$ is the interfacial tension of the surfactant-free interface, Γ is surfactant adsorption, α is the hard-disk area of the surfactant per mole (i.e., parking area; the reader is referred to [44] for a discussion about the different types of molecular areas), and *T* is temperature. The associated surface activity coefficient $f_{a}$ of the surfactant in the monolayer is:

(2) $$f_{a}=\frac{1}{1-\alpha \Gamma }{\left(\frac{2}{1+R_{\beta }}\right)}^{2+1/\beta }\exp\left[\frac{\alpha \Gamma (4-3\alpha \Gamma )}{{\left(1-\alpha \Gamma \right)}^{2}}\times \frac{2}{1+R_{\beta }}\right].$$

### 2.2. Hard-Disk Area α

For the SD model, the area parameter corresponds exactly to the cross-sectional area of the amphiphile molecule standing upright. Since the –OH group and the hydrocarbon chain are smaller than the perfluorocarbon chain, α is determined by the cross-sectional area of a perfluorocarbon chain, i.e., $\alpha =N_{A}\alpha_{{\mathrm{CF}}_{2}}$. The fluorocarbon chain has two preferential conformations: distorted antiperiplanar and helical [27,28], with different cross-sectional areas. Assuming a single constant hard-disk area for all studied surfactants, we previously found that $\alpha_{{\mathrm{CF}}_{2}}=24.5$ Å^2^, consistent with a helical conformation of the F-block, which agrees well with the tensiometric data for oil-soluble surfactants [25]. We will use this constant value throughout this paper as well, as an approximation. In reality, the cross-sectional area is a function of both the length of the blocks [29,30] and the temperature [28], and it has to be appreciated that the low molecular weight surfactants studied here may approach the area of the antiperiplanar configuration, $\alpha_{{\mathrm{CF}}_{2}}=21.6$ Å^2^, as the fluorocarbon chain length decreases. However, these 3 Å^2^ of difference result in a relatively small (approx. 5%) change in the transfer energy calculated below.

### 2.3. Attraction Parameter β

**Figure 1:**
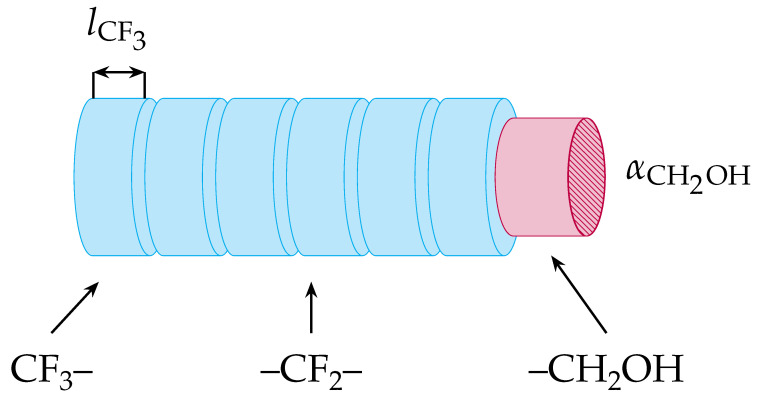
A fluorotelomer alcohol molecule modeled as a structure of connected cylindrical segments, as used in Ivanov’s $K_{a}$ model. Each segment is characterized by a height $l_{i}$, cross-sectional area $\alpha_{i}$, and a free energy of transfer of the segment $\Delta \mu_{i}$ from oil to water.

As we did previously, we calculate β based on a combination of osmotic attraction and effective Van der Waals interaction between the F-blocks through hexane [43]:

(3) $$\beta =\beta_{\mathrm{osm}}+\frac{1}{R_{{\mathrm{CF}}_{2}}^{2}}\int_{2R_{{\mathrm{CF}}_{2}}}^{\infty }\left\{\exp\left[\frac{nL_{{\mathrm{CF}}_{2}}}{4RTl_{{\mathrm{CF}}_{2}}r^{5}}\left(\frac{nl_{{\mathrm{CF}}_{2}}r}{r^{2}+n^{2}l_{{\mathrm{CF}}_{2}}^{2}}+3\arctan\frac{nl_{{\mathrm{CF}}_{2}}}{r}\right)\right]-1\right\}rdr,$$

where $l_{{\mathrm{CF}}_{2}}=1.306$ Å [45] is the height of the –CF_2_– fragment, and $R_{{\mathrm{CF}}_{2}}=\sqrt{\alpha_{{\mathrm{CF}}_{2}}/\pi }=2.79$ Å is the effective radius of the –CF_2_– fragment (see Figure 1). The presence of solvent molecules in the monolayer results in the depletion contribution, which was found to be $\beta_{\mathrm{osm}}=0.17$ for perfluoroalkylated alcohols at water|hexane [25]. The parameter $L_{{\mathrm{CF}}_{2}}=6.32\times 10^{-54}$ J·m^6^/mol is an effective interaction constant for –CF_2_– groups trough hexane [25]. Equation 3 represents well the dependence of β on the number *n* of perfluorinated carbon atoms for longer, oil-soluble homologues [25]. However, the dependence of β on temperature extracted from the experimental data is somewhat steeper than the theoretical one, probably due to the neglected effect of the temperature on the hard-disk area α. For the studied surfactants of n=1,2,3 at 293 K, Equation (3) predicts the values β= 0.26, 0.51, and 0.94, respectively.

### 2.4. Adsorption Constant $K_{a}$

In general, the adsorption isotherm of the surfactant reads:

(4) $$K_{a}C=f_{a}\Gamma ,$$

assuming that the aqueous surfactant solution is ideal (that the surfactant’s bulk activity coefficient is unity). The surface activity coefficient $f_{a}$ is controlled by the intralayer interactions, through the area per molecule α (a measure of the repulsive interaction) and the attraction parameter β (a measure of the attractive interactions in the monolayer); see the SD model’s Equation (2). It should be noted that $f_{a}$ varies significantly with the adsorption model. In particular, it is different for theories with localized (site) and nonlocalized (two-dimensional fluid) adsorption. The third adsorption parameter appearing in the adsorption isotherm—the adsorption constant $K_{a}$—is a characteristic of the adsorption of a single molecule on the neat interface; therefore, the value of $K_{a}$ is independent of the adsorption model.

For long monoblock surfactants, the adsorption constant is an exponential function of the so-called adsorption free energy, $K_{a}=\delta_{a}\exp\left(-E_{a}/RT\right)$, where the adsorption energy $E_{a}$ corresponds to the minimum of the free energy of the surfactant molecule at the surface [14] and is a linear function of the chain length *n*. The pre-exponential factor $\delta_{a}$ is known as the adsorption length (due to its dimensions), and it is common to assume that it is equal to the length of the surfactant molecule, as suggested by Davies [14]. However, Ivanov et al. [41] showed that the actual value of $\delta_{a}$ is about an order of magnitude lower than the size of the surfactant [22,41] and does not depend on the chain length. The theory of Ivanov et al. has been shown to predict adequate values for $K_{a}$ for water-soluble alkyl-based nonionic and ionic surfactants, both at water|air and water|oil [22,46], and also for the effective adsorption constant from water to a monolayer in the liquid expanded phase [21,46].

Ivanov’s model predicts deviation from the simple exponential (Traube-like) dependence $K_{a}\left(n\right)$ for very short surfactants—there, a more complex dependence is expected:

(5) $$K_{a}=\delta_{a}\left[1-\exp\left(-n\frac{\Delta \mu_{{\mathrm{CF}}_{2}}}{RT}\right)\right]\exp\left(-\frac{E_{a}\left(n\right)}{RT}\right)-nl_{{\mathrm{CF}}_{2}}.$$

This is a variant of Equation (2.15) from ref. [46]. For long-chained surfactants, Equation (5) simplifies to Davies’ expression: $K_{a}=\delta_{a}\exp\left(-E_{a}\left(n\right)/RT\right)$. However, for *n* equal to 1 or 2 (which are of interest here), the more complicated dependence on the size of the chain *n* has to be used, i.e., Equation (5).

Ivanov’s model has also been generalized to the adsorption constant of diblock surfactants [25]: as in the original model, the molecule is represented as a stack of cylindrical segments of different lengths and different free energy penalties for transfer from oil to water (see Figure 1). However, the alpha carbon next to the –OH group has been found to behave as part of the polar group that remains immersed in the aqueous phase [15,22]. Therefore, for the fluorotelomer alcohols studied here, the hydrophobic tail is a fluorocarbon, and Equation (5) is sufficient. In this case, the adsorption length is:

(6) $$\delta_{a}=\frac{RTl_{{\mathrm{CF}}_{2}}}{2\Delta \mu_{{\mathrm{CF}}_{2}}},$$

and the adsorption free energy from water to water|oil is:

(7) $$E_{a}=-\frac{2\sqrt{3}}{\pi }\alpha \gamma_{0}-\left(n-1\right)\Delta \mu_{{\mathrm{CF}}_{2}}-\Delta \mu_{{\mathrm{CF}}_{3}},$$

where $\Delta \mu_{{\mathrm{CF}}_{2}}$ and $\Delta \mu_{{\mathrm{CF}}_{3}}$ are the free energy changes of transfer from oil to water of the –CF_2_– and –CF_3_ moieties, respectively. The interfacial tension of neat water|hexane is a linear function of the temperature: $\gamma_{0}/\left[\mathrm{mN}/m\right]=50.56-0.0876\left(T/\left[K\right]-298.15\right)$ (an average from literature data [47,48,49,50,51]). The first term in Equation (7) is the interfacial energy gained by removing the $2\sqrt{3}/\pi \times \alpha$ contact area between hexane and water, when a surfactant molecule is adsorbed. The coefficient $2\sqrt{3}/\pi$ is the ratio between the hard-disk area and the partial area per surfactant molecule in the mixed monolayer of water and surfactant, assuming a quasi-hexagonal order. The second and third terms stand for the removal of the fluorocarbon chain|water contact area and the creation of a fluorocarbon chain|hexane contact area. For hydrocarbons, there is evidence that the $\Delta \mu_{{\mathrm{CH}}_{3}}/\Delta \mu_{{\mathrm{CH}}_{2}}$ is equal to the ratio of the contact areas with water for both moieties, as it follows from the hydrophobic/entropic origin of the transfer energies $\Delta \mu_{{\mathrm{CH}}_{2}}$ and $\Delta \mu_{{\mathrm{CH}}_{3}}$, i.e., $\Delta \mu_{{\mathrm{CH}}_{3}}/\Delta \mu_{{\mathrm{CH}}_{2}}\approx 2$ [22]. For fluorocarbons, even though the fluorine atom is bigger, the contact area of the CF_3_– moiety with water is also approximately twice as large as that of the –CF_2_– moiety. Therefore, in the present report, we use the approximation $\Delta \mu_{{\mathrm{CF}}_{3}}=2\Delta \mu_{{\mathrm{CF}}_{2}}$. This leaves only a single unknown in the adsorption model: the sought value of the transfer energy, $\Delta \mu_{{\mathrm{CF}}_{2}}$.

## 3. Results and Discussion

**Figure 2:**
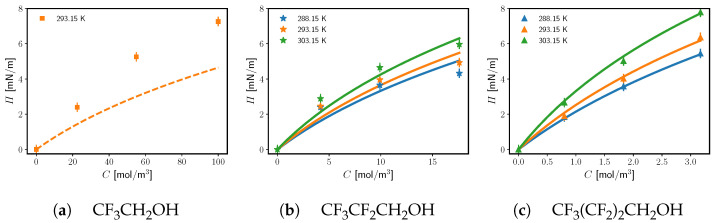
The interfacial pressure as a function of surfactant concentration in the aqueous phase at different temperatures for the three alcohols. Points are experimental data; lines are the SD model combined with the molecular thermodynamic Equations (1)–(5) for the adsorption parameters, with a single fitting parameter $\Delta \mu_{{\mathrm{CF}}_{2}}$. The fitting is done on the data for CF_3_CF_2_CH_2_OH and ${\mathrm{CF}}_{3}{\left({\mathrm{CF}}_{2}\right)}_{2}{\mathrm{CH}}_{2}\mathrm{OH}$ at each temperature, while CF_3_CH_2_OH is omitted, since Ivanov’s $K_{a}$ model deviates from the data for the shortest homologue (see the text).

The experimental results for the interfacial pressure as a function of the alcohol concentrations are presented in Figure 2. The increase in temperature results in an increase of the interfacial pressure, i.e., the adsorption of aqueous perfluoroalkylated surfactants to the water|oil interface is an endothermic process. This is in contrast to the adsorption of longer oil-dissolved fluorotelomer alcohols to water|oil, e.g., ${\mathrm{CF}}_{3}{\left({\mathrm{CF}}_{2}\right)}_{6}{\mathrm{CH}}_{2}\mathrm{OH}$ studied by Takiue et al. [32], which is exothermic.

**Figure 3:**
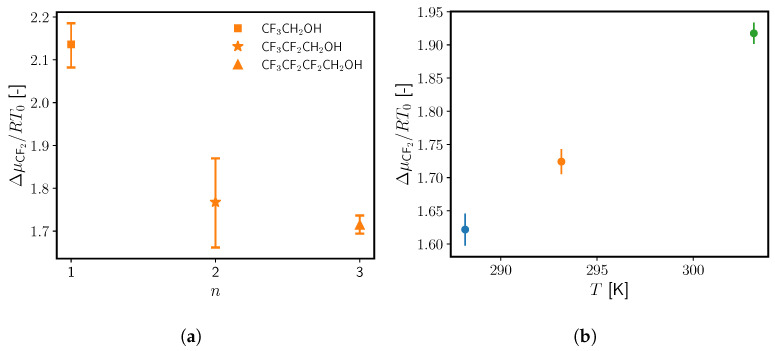
(**a**) Average free energy of transfer $\Delta \mu_{{\mathrm{CF}}_{2}}$ calculated from each experimental surface tension at 293.15 K independently. The error bars specify the lowest and highest calculated $\Delta \mu_{{\mathrm{CF}}_{2}}$. The data for n=1 (CF_3_CH_2_OH) deviate significantly from the rest and are excluded from the following analysis. (**b**) Best fit value of the free energy of transfer $\Delta \mu_{{\mathrm{CF}}_{2}}$ from simultaneous regression over the data for CF_3_CF_2_CH_2_OH and ${\mathrm{CF}}_{3}{\left({\mathrm{CF}}_{2}\right)}_{2}{\mathrm{CH}}_{2}\mathrm{OH}$ as a function of the temperature. The different temperatures are color-coded in accordance with Figure 2.

As a preliminary analysis, we calculated a $\Delta \mu_{{\mathrm{CF}}_{2}}$ value from each experimental surface pressure Π. This is done by calculating from Equation (1) the value of the adsorption Γ, using Equation (2) to calculate the respective activity coefficient $f_{a}$, employing Equation (4) to calculate $K_{a}$, and finally solving Equations (5) and (7) for $\Delta \mu_{{\mathrm{CF}}_{2}}$. The averaged results are presented in Figure 3a. From the graph, it would appear that there are two populations of data: CF_3_CH_2_OH produces values scattered around $2.15\times RT_{0}$, while the CF_3_CF_2_CH_2_OH and $\mathrm{CF}3{\left({\mathrm{CF}}_{2}\right)}_{2}{\mathrm{CH}}_{2}\mathrm{OH}$ data are scattered around $1.8\times RT_{0}$. The confidence intervals of the latter two overlap, while the distance of the fluorinated ethanol cluster from the fluorinated propanol and butanol cluster is more than twice the deviation within a dataset for a single surfactant. The average value of $\Delta \mu_{{\mathrm{CF}}_{2}}$ obtained for CF_3_CH_2_OH is not only higher than that obtained from CF_3_CF_2_CH_2_OH and ${\mathrm{CF}}_{3}{\left({\mathrm{CF}}_{2}\right)}_{2}{\mathrm{CH}}_{2}\mathrm{OH}$, but also rather high compared to previous estimates of $\Delta \mu_{{\mathrm{CF}}_{2}}$ [25]. Therefore, we can conclude that the results for CF_3_CH_2_OH are qualitatively different than the other two alcohols. In the literature, there are other examples where the first member of a homologous series behaves differently from the rest, e.g., the closely related solvation free energy of hydrocarbons in water [52]. It is reasonable to assume that the $K_{a}$ model (Equation (5)) is inadequate for n=1. A likely reason for this is the interaction between the polar –CF_3_ group and the aqueous phase, which may produce a more horizontal orientation of the adsorbed molecules. This violates the assumptions of Ivanov’s model. Therefore, for CF_3_CH_2_OH, (i) the 1/2 factor in Equation (6) for the adsorption length should be reevaluated, and (ii) the area per molecule should be larger than $\alpha_{{\mathrm{CF}}_{2}}$. Hence, we exclude the fluorinated ethanol from the following analysis.

To calculate the transfer energy $\Delta \mu_{{\mathrm{CF}}_{2}}$ with higher accuracy, we fit simultaneously all experimental points for a temperature with a single $\Delta \mu_{{\mathrm{CF}}_{2}}$. The Levenberg–Marquardt algorithm is used to minimize the root-mean-square deviation between the model predictions and the experimental surface pressures. Since CF_3_CH_2_OH is excluded from the fitting procedure, each temperature has six experimental points coming from the propanol and butanol, i.e., a single parameter fit to six points from two homologues. The obtained optimal $\Delta \mu_{{\mathrm{CF}}_{2}}$ values are presented in Table 2. The error of the fitted $\Delta \mu_{{\mathrm{CF}}_{2}}$, as determined from the root of its variance, is very low: $0.02RT_{0}$ (ca. 50 J/mol).

The optimal root-mean-square deviations between the experimental and the theoretical Π are 0.6 mN/m and 0.1 mN/m for CF_3_CF_2_CH_2_OH and ${\mathrm{CF}}_{3}{\left({\mathrm{CF}}_{2}\right)}_{2}{\mathrm{CH}}_{2}\mathrm{OH}$, respectively (see Figure 2). For ${\mathrm{CF}}_{3}{\left({\mathrm{CF}}_{2}\right)}_{2}{\mathrm{CH}}_{2}\mathrm{OH}$, the deviation 0.1 mN/m is less than the experimentally determined reproducibility. The higher deviation for CF_3_CF_2_CH_2_OH reflects the fact that the experimental points do not neatly fit the SD model (see Figure 2). This is assumed to be due to surface active impurities, up to 3% as reported by the vendor. On the other hand, since the butanol is about five times more surface active as compared to propanol, the impurities in ${\mathrm{CF}}_{3}{\left({\mathrm{CF}}_{2}\right)}_{2}{\mathrm{CH}}_{2}\mathrm{OH}$ have a much smaller effect, thus, the lower deviation. Fortunately, the uncertainty in the tensiometric measurement propagates logarithmically to the transfer energy $\Delta \mu_{{\mathrm{CF}}_{2}}$. Therefore, the effect of the impurities in CF_3_CF_2_CH_2_OH is small in terms of the uncertainty of the obtained transfer energy $\Delta \mu_{{\mathrm{CF}}_{2}}$.

A linear relationship between $\Delta \mu_{{\mathrm{CF}}_{2}}$ and *T* is observed (Figure 3b). The free energy change is comprised of an entropic and an enthalpic term:

(8) $$\Delta \mu_{{\mathrm{CF}}_{2}}=\Delta h_{{\mathrm{CF}}_{2}}-T\Delta s_{{\mathrm{CF}}_{2}}.$$

Assuming that both are temperature independent, from the results in Figure 3, the enthalpy for transfer of a –CF_2_– group from hexane to water can be determined as $\Delta h_{{\mathrm{CF}}_{2}}=-10.0$ kJ/mol and the respective entropy as $\Delta s_{{\mathrm{CF}}_{2}}=-48.7$ J/mol K. The obtained value of $\Delta s_{{\mathrm{CF}}_{2}}$ is larger than expected (compare to methylene’s $\Delta s_{{\mathrm{CH}}_{2}}$ on the order of −10 J/mol K [52]). At this length scale, the hydrophobic effect is approximately proportional to the surface area of the segment [53]. Therefore, $\Delta s_{{\mathrm{CF}}_{2}}$ is expected to be about 1.3 times higher than $\Delta s_{{\mathrm{CH}}_{2}}$. However, we determine an entropy that is about 5 times higher than $\Delta s_{{\mathrm{CH}}_{2}}$. This discrepancy might be due to the assumptions of our model, in particular about the temperature dependence of the adsorption parameters. For example, α, which appears in both Equation (7) for $K_{a}$ and the equation of state (Equation (1)), is assumed to be independent of *T*, which would result in a compensation by an artificial variation of $\Delta \mu_{{\mathrm{CF}}_{2}}$ with *T* and, respectively, an unrealistic value of $\Delta s_{{\mathrm{CF}}_{2}}$. Nevertheless, the effect on the resultant $\Delta \mu_{{\mathrm{CF}}_{2}}$ values appears to be relatively small: even if we used $\Delta s_{{\mathrm{CF}}_{2}}=1.3\times \Delta s_{{\mathrm{CH}}_{2}}$, for the current narrow temperature interval, the error introduced in $\Delta \mu_{{\mathrm{CF}}_{2}}$ would be within 5% of the values in Figure 3b. The enthalpy $\Delta h_{{\mathrm{CF}}_{2}}$ is also higher than the corresponding $\Delta h_{{\mathrm{CH}}_{2}}$, which is close to zero at room temperature [21]. This could be justified physically with the fact that, unlike –CH_2_– chains, for –CF_2_– chains, there is a significant Van der Waals contribution to $\Delta \mu_{{\mathrm{CF}}_{2}}$ (the dispersion contribution to β as studied in ref. [25]).

## 4. Materials and Methods

All reagents—hexane (C_6_H_14_, ≥99%), 2,2,2-trifluoro-1-ethanol (CF_3_CH_2_OH, ≥99%), 2,2,3,3,3-pentafluoro-1-propanol (CF_3_CF_2_CH_2_OH, ≥97%), and 2,2,3,3,4,4,4-heptafluoro-1-butanol (CF_3_CF_2_CF_2_CH_2_OH, ≥98%)—were purchased from Sigma-Aldrich and used without further purification. All water used was double distilled using a GFL 2001/2 distiller. Aqueous solutions close to the expected solubility limit were prepared volumetrically and subsequently diluted approximately two and four times (the exact concentrations are reported in Figure 2).

The interfacial tension of each solution was measured with a profile analysis tensiometer (PAT-1, Sinterface, Germany). A hexane drop with constant area, ca. 25 mm^2^, was formed in a 25 cm^3^ glass cuvette filled with the aqueous solution. The cuvette and the capillary were cleaned before each experiment by immersion in a dichromate solution for at least 24 h. The temperature of the measuring cell was controlled precisely with a thermostat (Ecoline E200, LAUDA DR. R. WOBSER GMBH & CO. KG, Lauda-Königshofen, Germany). Measurements were made at 288.15 K, 293.15 K, and 303.15 K. In a typical experiment, the interfacial tension was observed for ca. 6 h at a constant temperature so as to ensure that the value had settled. Within the studied concentration range, the solution density is assumed to be constant [54]. The average standard deviation of the measured interfacial tension determined from multiple repetitions is 0.26 mN/m.

## 5. Conclusions

We have determined experimentally the free energy of transfer of a –CF_2_– group from oil to water $\Delta \mu_{{\mathrm{CF}}_{2}}$ from data for the adsorption of water-soluble nonionic fluorosurfactants at water|hexane. As far as we are aware, this has been done for the first time, since only the shortest nonionic fluorinated surfactants (*n* = 1–3) are water-soluble. The values obtained are $1.68\pm 0.02\times RT_{0}$, $1.75\pm 0.02\times RT_{0}$, and $1.88\pm 0.02\times RT_{0}$ at 288.15 K, 293.15 K, and 303.15 K, respectively, and appear to be of lower uncertainty compared to previous reports [25].

The short length of the fluorocarbon chain results in complications—in particular, the deviations from Traube’s rule must be taken into account. While we were successful in resolving this issue for n≥2 by using Ivanov’s explicit model for $K_{a}\left(n\right)$, we found that the adsorption data for the first homologue CF_3_CH_2_OH suggest a very different behavior from the rest of the series.

The obtained value of $\Delta \mu_{{\mathrm{CF}}_{2}}$ is within the expected range (see the discussion in Peychev and Slavchov [25]). As it can be deduced from the more hydrophobic behavior of fluorocarbons compared to hydrocarbons, the value of $\Delta \mu_{{\mathrm{CF}}_{2}}$ is higher than the corresponding free energy of transfer of a –CH_2_– group from oil to water ($\Delta \mu_{{\mathrm{CH}}_{2}}=1.39\times RT_{0}$). However, the result at 293.15 K, $\Delta \mu_{{\mathrm{CF}}_{2}}=1.75\pm 0.02\times RT_{0}$, is lower than the value $2.05\times RT_{0}$ reported by Mukerjee and Handa [24], based on similar tensiometric experiments but with fluorinated ionic surfactants. Due to the exponential relationship between $K_{a}$ and $n\Delta \mu_{{\mathrm{CF}}_{2}}$, this is a very large difference. For instance, for n=3, the adsorption constant predicted from Equation (5) with Mukerjee and Handa’s value $\Delta \mu_{{\mathrm{CF}}_{2}}=2.05\times RT_{0}$ is twice as high as what we measured experimentally.

The obtained result for $\Delta \mu_{{\mathrm{CF}}_{2}}$ should be useful for prediction of the partitioning of mixtures of short fluorinated amphiphiles between water and oil and between water and biomembranes [13], through the respective partition coefficient, $RT\ln K_{p}\propto n\Delta \mu_{{\mathrm{CF}}_{2}}$ [25,55]. The transfer energy is also an essential parameter for predicting the incorporation of water-dissolved fluorinated amphiphiles into micelles and into adsorption monolayers made of hydrocarbon surfactants (compare to the effective adsorption constant of a liquid expanded monolayer [21,46]). These fundamental thermodynamic characteristics are essential for understanding the environmental fate of perfluoroalkyl substances and their health effects and for modeling separation processes that involve them.

## Figures and Tables

**Table 1:** The investigated fluoroalcohols.

*n*	Formula	Name
1	CF_3_CH_2_OH	2,2,2-trifluoro-1-ethanol
2	CF_3_CF_2_CH_2_OH	2,2,3,3,3-pentafluoro-1-propanol
3	CF_3_CF_2_CF_2_CH_2_OH	2,2,3,3,4,4,4-heptafluoro-1-butanol

**Table 2:** Results for the optimized transfer energy $\Delta \mu_{{\mathrm{CF}}_{2}}$ and the values of the corresponding enthalpy $\Delta h_{{\mathrm{CF}}_{2}}$ and entropy $\Delta s_{{\mathrm{CF}}_{2}}$.

*T* [K]	$\Delta {\mu_{\mathrm{CF}}}_{2}/{\mathrm{RT}}_{0}$ [-]
288.15	1.68±0.02
293.15	1.75±0.02
303.15	1.88±0.02
$\Delta h_{{\mathrm{CF}}_{2}}$ [kJ/mol]	$\Delta s_{{\mathrm{CF}}_{2}}$ [J/mol K]
−10.0±0.2	−48.7±0.7

## Data Availability

Dataset available on request from the authors.
